# Nonlinear stability of two dusty magnetic liquids surrounded via a cylindrical surface: impact of mass and heat spread

**DOI:** 10.1038/s41598-023-33025-1

**Published:** 2023-05-01

**Authors:** Galal M. Moatimid, D. M. Mostafa

**Affiliations:** Department of Mathematics, Faculty of Education, Ain Shams University, Roxy, Cairo, Egypt

**Keywords:** Energy science and technology, Engineering, Mathematics and computing, Physics

## Abstract

The current article examines a nonlinear axisymmetric streaming flow obeying the Rivlin–Ericksen viscoelastic model and overloaded by suspended dust particles. The fluids are separated by an infinite vertical cylindrical interface. A uniform axial magnetic field as well as mass and heat transmission (MHT) act everywhere the cylindrical flows. For the sake of simplicity, the viscous potential theory (VPT) is adopted to ease the analysis. The study finds its significance in wastewater treatment, petroleum transport as well as various practical engineering applications. The methodology of the nonlinear approach is conditional primarily on utilizing the linear fundamental equations of motion along with the appropriate nonlinear applicable boundary conditions (BCs). A dimensionless procedure reveals a group of physical dimensionless numerals. The linear stability requirements are estimated by means of the Routh–Hurwitz statement. The application of Taylor’s theory with the multiple time scales provides a Ginzburg–Landau equation, which regulates the nonlinear stability criterion. Therefore, the theoretical nonlinear stability standards are determined. A collection of graphs is drawn throughout the linear as well as the nonlinear approaches. In light of the Homotopy perturbation method (HPM), an estimated uniform solution to the surface displacement is anticipated. This solution is verified by means of a numerical approach. The influence of different natural factors on the stability configuration is addressed. When the density number of the suspended inner dust particles is less than the density number of the suspended outer dust particles, and vice versa, it is found that the structure is reflected to be stable. Furthermore, as the pure outer viscosity of the liquid increases, the stable range contracts, this means that this parameter has a destabilizing effect. Additionally, the magnetic field and the transfer of heat don’t affect the nature of viscoelasticity.

## Introduction

Magnetic fluids have more different fluid dynamics than the conventional fluid in that magnetic stress appears, and, unlike MHD, electrical currents were required^1^. It developed a stress tensor expression for magnetic fluids with an arbitrary single-valued magnetization. A magnetic fluid may be characterized as Ferrofluid, which was a colloidal combination made up of nanoscale ferromagnetic particles (10 nm) covered with a surfactant to avoid accumulation and dispersed in a fluid carrier, commonly an organic solvent or water^2^. Magnetic fluids have attracted a lot of attention when they were subjected to tangential and radial magnetic fields. The nonlinear wave propagation of the free surface of a magnetic liquid when it was revealed to have an inclined magnetic strength was designed^3^. Some illustrations showed macroscopic Ferrofluid disturbances which resulted in designs, lines, and developments that can then be condensed in size to sub-micron dimensions^4^. A number of advances have been designed to reach medications to a particular part of the human body via lipid nanoparticles and it released red blood cells. Magnetic fluid**s** compress huge potential applications and can be best exploited in both fields of biotechnology and biomedical sciences^5^. The development of the oscillating Marangoni effect was studied via the linear stability theory^6^. A heat gradient and a high-incline magnetic strength were used to investigate the linear stability of parallel flows^7^. The stability analysis revealed that the magnetic Rayleigh number was unaffected by the magnetic field inclination, however, the crucial gravitational Rayleigh numeral changed somewhat with some deviations. The instability of wave propagation separating two immiscible magnetic liquids was investigated^8^. Analytically and graphically, various stability requirements were addressed. Short waves below the essential wave numbers were found to be stable; however, a number of long waves were discovered to be unstable. The stability of straightforward flowing magnetic liquids in the existence of an incline magnetic strength was investigated^9^. The methodology used the linear analysis of improved Kelvin–Helmholtz instability (KHI) with the effect of MHT through the interface. It was revealed that the significance of the field was contingent deeply on the selection of a few basic factors of the structure. A novel mathematical method for the KHI saturation in permeable media with MHT was investigated in Refs.^10,11^. Two limited horizontal magnetic liquids were operated upon by a uniform tangential magnetic field. A consistent axial magnetic field affects the structure, where the existence of surface currents at the surface of separation was enabled by the magnetic field intensities. In view of the extensive products in diverse areas of magnetic fluids, the current work examines the interface stability connecting two different magnetic liquids.

The phenomena of double-diffusive convective in an elastic viscous nanofluid were essential in electrochemistry, food manufacturing, engineering and nuclear technologies, geophysics, bioengineering cancer therapy, physiological fluid motion, oceanography, and many other domains. Oldroyd^12^ developed the constitutive equations of fluids and called them Oldroyd fluids. It displayed that the key non-Newtonian flow features are found in moving polymer solutions and other elastic-viscous liquids. Chandrasekhar^13^ investigated a large number of thermal instability problems, concerning Newtonian and non-Newtonian fluids in the hypotheses of Hydrodynamics and Hydromagnetic. The Rayleigh–Taylor instability (RTI) of a Newtonian viscous liquid above a Rivlin–Ericksen viscoelastic fluid with suspended particles in a permeable medium was investigated^14^. The influence of Hall currents on the convective stability of a Rivlin–Ericksen fluid in a permeable medium under the influence of a steady normal magnetic field was considered^15,16^. The inspiration for this work came from the reality of the technological and engineering significance of fluid-particle combinations and the significance of suspended particles in the instability problems of superimposed viscoelastic liquids. Additionally, they were useful in a variety of geophysical and chemical applications. It was found that the stability standards were independent of the effects of viscosity and viscoelasticity and were dependent on the orientation and magnitude of the magnetic field. The influence of the quantum phenomena on the RTI in an endless stratification flow passing through a permeable medium was studied^17^. It was discovered that, in addition to the quantum impact, the vertical magnetic field provided so much stability to the system under consideration. The RTI of a viscoelastic Rivlin–Ericksen dusty liquid passing through a permeable medium in a strong magnetic field was studied^18,19^. The dispersion relation was calculated using linearized theory and normal mode analysis. The KHI suppressed the efficient interfacial tension for small wavelength perturbations, although considerable porosity limits the stability range expressed in terms of a variation in streaming velocities. The pressure-driven temporary Rivlin–Ericksen liquid and the irreversibility of varying viscosity on a hydromagnetic two-step exothermic chemical reacting flow through a convection cooling system were analyzed^20^. Lubrication firms will be interested in these if they intend to improve the effectiveness of hydromagnetic substances used in engineering systems. The examination of the Rivlin–Ericksen liquid was interesting due to the increasing impact of nanofluid in current sciences and technology. Therefore, the current work conducts an instability analysis of a cylindrical surface connecting two dusty Rivlin–Ericksen liquids.

Numerous stability problems have been examined utilizing the VPF. For liquids, VPF created a solution to the Navier–Stokes equation that has exactly zero vorticity. Nevertheless, in VPF, the viscous effects of stress in an incompressible liquid were not zero; hence the viscous term no longer exists. In addition, for VPF to meet the border requirements, the tangential component of velocity and shear stress should be continuous throughout the surface of the separation of the liquid from a substance or some other liquid. Balanced and tangential stresses were not considered in the VPF; only normal stress was used to establish the existence of viscosity. A study on the impact of suspended particles on the beginning of Bènard convection was performed^21^. Therefore, it was observed that the layer influence on the suspended particles was destabilizing. Another driving force behind this problem was the fact that knowing fluid-particle interactions did not align with their commercial interests. It is necessary to expand the instability zone of the stratified fluids with fine dust shear flow^22^. Joseph et al.^23^ examined the RTI of two viscous fluids employing VPF. Additionally, the VPF was expanded to incorporate the viscoelastic fluid RTI problem^24^. It was discovered that the critical wavelengths and growth rate provided by the viscoelastic potential theory were accurate. Lately, Joseph^25^ has provided an excellent examination of this problem. This review showed that all the theorems relating to the hypothetical flow of inviscid liquids with conservative body forces were equally applicable in viscous fluids across the regions of the irrotational flow. The boundary between two viscous layers of linear EHD instability with VPF was studied^26^. A number of graphs were employed to illustrate the effects of different factors on the stability profile. They observed that the stability pattern is stabilized by the permeable Darcy's factor. The stability of thin sheets of viscous and dielectric liquids was addressed by the application of the VPF^27^, where air viscosity destabilizes structures, while liquid viscosity stabilizes them. In chemistry and manufacturing engineering, the effect of suspended particles on the stability of superposed suspended liquids may have significant implications. Consequently, dusty liquids are considered in the current study given their significance. This principle is the foundation of the current program, but only if one agrees with how simple it is to establish a VPT agreement.

Nuclear waste disposal underground, filtration processes, and geothermal reservoirs are all examples of heat exchange convection in a porous material. For their wide variety of applications throughout many contexts, including boiling heat transmission in manufacturing as well as technology and geophysics difficulties, the MHT occurrence in multiphase movements has been considered in recent years. Hsieh^28,29^ established the general formulation of an interfacial flow issue of two inviscid fluids with heat and mass transfer for both RTI and KHI in plane geometry. Hsieh^28,29^ is considered the first scientist who adapted this simplified technique to investigate both linear and nonlinear stability problems. Ho^30^ considered the linear analysis of the RTI of two viscous liquids with the same kinematic viscosities in the existence of MHT. In the stability criterion, it was discovered that MHT played a stabilizing influence. The KHI has a destabilizing effect on stability and configuration, according to Nayak and Chakraborty^31^. Furthermore, the KHI was lower in flat geometry than in cylindrical geometry. The RTI and KHI of the liquid–vapor surface and the vapor to be flowing horizontally were investigated. It was discovered that coupled viscosity-phase transition has a stabilizing impact on RTI, but a destabilizing influence on KHI, and that these distributions depended on both the Marangoni effect and the surface tension. Despite the energy and concentration equations in the current article, the obtained results were in good agreement with those previously obtained^32^. There hasn't been any discussion of the Carreau viscosity model for a moving wedge with heat generation/absorption and chemically reactive species^33^. It was shown that the temperature profile was seen to increase for positive values while declining under the impact of negative values. With the Marangoni convection effect, Moatimid et al.^34^ examined these situations in a temporal instability of a restricted nano-liquid layer. It was impossible to overstate the importance of the commencement of convection in a horizontal nanofluid layer of finite depth since it is a key factor in regulating the diffusion phenomenon in a nanofluidic medium^35^. The nonlinear instability of a vertical cylindrical boundary separation between two streaming Reiner-Rivlin liquids was mathematically examined^36^. An unchanging longitudinal electric strength was used to represent the system. Additionally, the effects of MHT and permeable media were considered. The current work investigates the interface instability in the presence of MHT in light of the significance of MHT.

As aforementioned, Rivlin–Ericksen viscoelastic fluid has wide-ranging purposes in industry. The hydromagnetic stability of stratified Rivlin–Ericksen viscoelastic fluids in the presence of suspended particles across a permeable medium could be useful in modern technology. Several researchers have looked at this topic. However, the hydrodynamic stability of a stratified Rivlin–Ericksen viscoelastic fluid in the presence of suspended particles across a permeable medium has not been previously touched upon. Therefore, the current paper involves all these aspects. Rivlin–Ericksen fluid is inserted into circular vertical cylinders. The interior and exterior liquids are both exposed to an axial homogeneous longitudinal magnetic strength. In addition to methodological interest, the topic also has scientific as well as practical relevance. The stability conditions are determined and proven theoretically and quantitatively. Consequently, the estimated profile of the surface deflection solution is determined. We are motivated to investigate the stability of stratified viscoelastic Rivlin–Ericksen fluid in the presence of a uniform tangential magnetic field in the current paper because of the significance of non-Newtonian fluids in contemporary technology, industries, chemical engineering, and other fields. Additionally, the fluid is frequently not pure in geophysical settings and has suspended particles. The major objective of the current study is to encourage readers to look for appropriate answers to the following quires:

- What are the criteria of the linear stability approach?
- How many physical non-dimensional numbers are present throughout the linear performance?
- What is the influence of these dimensionless numbers on the phase diagram?
- What is the approach of the nonlinear stability sense?
- What is the estimated solution to the interface displacement?

The rest of the article is organized to understand the scheme presentation as follows: “Construction of the problem” introduces the problem formulation, which contains the underlying dusty equations of motion as well as the relevant nonlinear border requirements. This Section also contains the solution method, which is based on normal mode analysis, as well as the governing nonlinear characteristic equation. The method of solution is presented in "Method of solution". "Linear stability analysis" introduces the linear dispersion relationship and stability analysis. In addition, the linear stability analysis of the linear approach is carried out in this section. "Nonlinear stability analysis" discusses the nonlinear stability that emerges in a Ginzburg–Landau equation, as well as the theoretical and numerical calculations. In "Approximate interface displacement profile", a uniform approximate solution of the interface displacement is derived in the light of the HPM using the extended frequency idea. This Section also includes a numerical estimation of this distribution. In "Concluding remarks", the obtained results are reported as conclusions. The main conclusions about the effect of different material factors in the evaluation of linear/nonlinear instability of the problem at hand are given in this section.

## Construction of the problem

**Figure 1:**
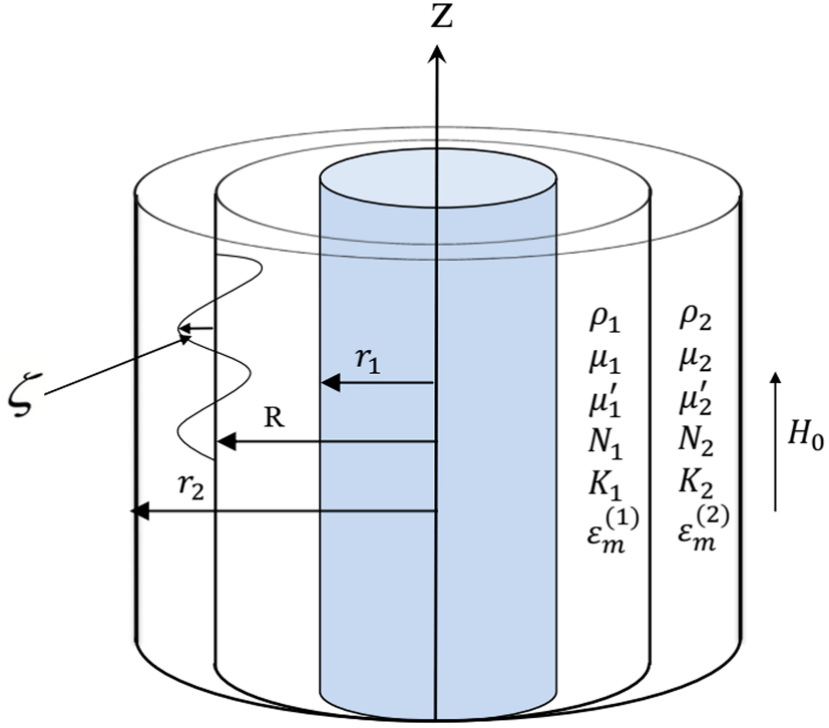
Sketches the theoretical model.

Two incompressible viscoelastic fluids of Rivlin-Ericksen type are restricted, connecting two solid cylinders in the existence of suspended dust particles of a number of different densities. Both an unchanged axial magnetic strength pervaded in the two media and MHT across the cylindrical interface exist. The pure liquids in the annular zone have different densities, viscoelasticities, viscosities, and magnetic permeabilities. Considering Hsieh’s simplified formulation^28,29^, the heat in the inner surface and outer cylinders is different. In light of the dusty particles, there are velocities and density numbers of the dust particles. One may assume that $\underline{U} (r,z;t)$ is the velocity vector of pure liquid. The coefficient of stokes drag is also considered. The hypothetical prototype of the structure at hand is drawn in Fig. 1

The fundamental equations of motion may be found in Refs.^37,38^ as follows:

1 $$ \rho \left( {\frac{\partial }{\partial t} + (\underline{U} .\nabla )} \right)\underline{U} = - \nabla P - \rho g\underline{e}_{z} - \frac{1}{{k_{1} }}\left( {\mu + \mu^{\prime}\frac{\partial }{\partial t}} \right)\,\underline{U} + KN(\underline{V} - \underline{U} ), $$

and

2 $$ \nabla .\underline{\,U} = 0. $$

The equations of motion and continuity for the dust particles, in which $mN$ is the particle mass unit volumes and $V$, indicate the suspended dust particles given by Sharma and Sharma^38^:

3 $$ {\text{K}}N(\underline {\text{U}} - \underline {V} ) - \mu \underline {V} = 0, $$

and

4 $$ \frac{\partial N}{{\partial t}} + \nabla \,.\,(N\underline{V} ) = 0. $$

When embedded dust particles are present, an additional force term corresponding to the speed differential equation connecting the dust particles and the liquid exists in Eq. (1). Regarding the particles, the buoyancy force is ignored. The relationships between particles are not considered, and we presume that particle distances are very huge in comparison to their diameter. The change in particle concentration is ignored because the numeral density of dust particles is considered to be modest. Therefore, Eq. (4) is not employed subsequently, and Eq. (1) becomes:

5 $$ \rho \left( {\frac{\partial }{\partial t} + (\underline{U} .\nabla )} \right)\underline{U} = - \nabla P - \rho g\underline{e}_{z} - \frac{1}{{k_{1} }}\left( {\mu + \mu^{\prime}\frac{\partial }{\partial t}} \right)\,\underline{U} - M\underline{U} , $$

where $$M = \frac{KN\mu }{{KN + \mu }}$$.

Considering a slight departure from the fundamental state of equilibrium, the interfacial profile $S(r,z;t)$ may be formulated as:

6 $$ S(r,z;t) = r - R - \zeta (z,t) = 0, $$

in which

7 $$ \underline{n} = \frac{\nabla S}{{\left| {\nabla S} \right|}} = (\underline{e}_{r} - \,\zeta_{z} \,\underline{e}_{z} )(1 + \zeta_{z}^{2} )^{ - 1/2} . $$

Utilizing Bernoulli's equation, pressure can be represented as follows:

8 $$ P_{j} = - \rho_{j} \left( {\frac{{\partial \phi_{j} }}{\partial t} + ikU_{0j} \phi_{j} } \right) - \frac{1}{{k_{1} }}\left( {\mu_{j} + \mu^{\prime}_{j} \frac{\partial }{\partial t}} \right)\phi_{j} - \left( {\frac{{K_{j} N_{j} \mu_{j} }}{{K_{j} N_{j} + \mu_{j} }}} \right)\phi_{j} ,\quad j = 1,2. $$

Our study is based on the VPT; hence the velocity of a pure fluid may be written as the gradient of the velocity distribution $\phi_{j} (r,z;t)$,where

$$ \underline{U}_{j} = U_{0j} \underline{e}_{z} - \nabla \phi_{j} = - \frac{{\partial \phi_{j} }}{\partial r}\underline{e}_{r} + \left( {U_{0j} - \frac{{\partial \phi_{j} }}{\partial z}} \right)\underline{e}_{z} . $$

The method of solution will be presented throughout the next section.

## Method of solution

Each perturbed physical quantity may be represented as follows:

9 $$ F\left( {r,z,t} \right) = \tilde{F}(r,t)e^{ikz} + c.c. $$

Here $\tilde{F}(r,t)$ is a random distribution of $r,t$.

The analysis assumes that both liquids are irrotational and incompressible, which leads to the velocity potential functions satisfying the Laplace equations. i.e.,

10 $$ \nabla^{2} \phi_{j} = 0. $$

In which $\nabla^{2} \equiv \frac{{\partial^{2} }}{{\partial r^{2} }} + \frac{1}{r}\frac{\partial }{\partial r} + \frac{{\partial^{2} }}{{\partial z^{2} }}$,and

11 $$ \phi_{j} \left( {r,z,t} \right) = \tilde{\phi }_{j} e^{ikz} + c.c. $$

Therefore, one gets:

12 $$ \tilde{\phi }_{j} (r;t) = C_{1}^{(j)} (t)I_{0} (kr) + C_{2}^{(j)} (t)K_{0} (kr), $$

where the time-dependent arbitrary functions are $C_{1}^{(j)} (t)\,\,{\text{and}}\,\,C_{2}^{(j)} (t).$

In our analysis, the estimate quasi-static is valid, therefore, the magnetic strength may be obtained as follows:

13 $$ H_{j} = H_{0} \underline{e}_{z} - \nabla \psi_{j} = - \frac{{\partial \psi_{j} }}{\partial r}\underline{e}_{r} + \left( {H_{0} - \frac{{\partial \psi_{j} }}{\partial z}} \right)\underline{e}_{z} . $$

Gauss's law requires that the potential function be:

14 $$ \nabla^{2} \psi_{j} = 0. $$

According to Eq. (9), the magnetic potential function can be expressed as:

15 $$ \tilde{\psi }_{j} (r;t) = C_{3}^{(j)} (t)I_{0} (kr) + C_{4}^{(j)} (t)K_{0} (kr), $$

where the time-dependent arbitrary functions are $C_{3}^{(j)} (t)\,\,{\text{and}}\,\,C_{4}^{(j)} (t).$

### Nonlinear boundary conditions

The speed and magnetic potential profiles, as presented in Eqs. (12) and (15), are subjected to the following conditions:

(i) On the rigid boundary $(r = r_{j} ,\,\,j = 1,2)$, the normal velocities of the fluid and the normal components of the potential should disappear; the corresponding boundary conditions are:
  16 $$ \left. \begin{gathered} \frac{{\partial \phi_{j} }}{\partial r} = 0 \hfill \\ \frac{{\partial \psi_{j} }}{\partial r} = 0 \hfill \\ \end{gathered} \right\}\,\,{\text{at }}\, \, r = r_{j} ,\,\,j = 1,2. $$
(ii) The tangential components of the magnetic displacements necessitate:
  17 $$ \underline{n} \wedge \left\| {\underline{H} } \right\| = 0,\quad {\text{at}}\quad r = R + \zeta \left( {z;t} \right), $$
  where $\left\| \bullet \right\| = \bullet_{2} - \bullet_{1}$ indicate the exterior and interior liquid jump, correspondingly.
(iii) The continuity of the normal magnetic induction components at the interface $r = R + \zeta \left( {z;t} \right)$ yields
  18 $$ \underline{n} \,.\,\left\| {\varepsilon_{m} \,\underline{H} } \right\| = 0. $$
(iv) The preservation of mass and energy through the perturbed surface requires^29^
  19 $$ \left\| {\rho \left( {\frac{\partial S}{{\partial t}} + \nabla \phi \cdot \nabla S} \right)} \right\| = 0. $$
(v) The energy at the perturbed surface^29^ requires:
  20 $$ \tilde{L}\rho_{1} \left( {\frac{\partial S}{{\partial t}} + \nabla \phi_{1} \cdot \nabla S} \right) = f\left( \zeta \right), $$
  where $f\left( \zeta \right)$ represents the net temperature flux. In the equilibrium situation, the temperature fluxes in the positive direction of $r$ in the fluid phases 1 and 2 are $- \kappa_{1} \left( {T_{1} - T_{0} } \right)/R\,Log\left( {r_{1} /R} \right)$ and $- \kappa_{2} \left( {T_{0} - T_{2} } \right)/R\,Log\left( {R/r_{2} } \right)$. As in Ref.^31^, we write:
  21 $$ f\left( \zeta \right) = \frac{{\kappa_{2} \left( {T_{0} - T_{2} } \right)}}{{\left( {R + \zeta } \right)\left( {Log\,r_{2} - Log\,\left( {R + \zeta } \right)} \right)}} - \frac{{\kappa_{1} \left( {T_{1} - T_{0} } \right)}}{{\left( {R + \zeta } \right)\left( {Log\,\left( {R + \zeta } \right) - Log\,r_{1} } \right)}}. $$

On expanding it by using Taylor's theory, we have

22 $$ f\left( \zeta \right) = f\left( 0 \right) + \zeta \,f^{\prime}\left( 0 \right) + \frac{1}{2}\zeta^{2} \,f^{\prime\prime}\left( 0 \right) + \frac{1}{3!}\zeta^{3} \,f^{\prime\prime\prime}\left( 0 \right) + \cdots , $$

we assume that $f\left( 0 \right) = 0,$ hence

23 $$ \frac{{\kappa_{2} \left( {T_{0} - T_{2} } \right)}}{{R\,Log\left( {r_{2} /R} \right)}} = \frac{{\kappa_{1} \left( {T_{1} - T_{0} } \right)}}{{R\,Log\left( {R/r_{1} } \right)}} = G, $$

where $G$ is steady, displaying that the temperature flux is equal through the perturbed surface connecting the two liquids in the equilibrium state.

From Eqs. (6), (20) and (22), one gets:

24 $$ \rho_{1} \left( { - \frac{\partial \zeta }{{\partial t}} - \frac{{\partial \phi_{1} }}{\partial r} + \frac{\partial \zeta }{{\partial z}}\left( {\frac{{\partial \phi_{1} }}{\partial z} - U_{01} } \right)} \right) = \alpha_{1} \left( {\zeta + \alpha_{2} \zeta^{2} + \alpha_{3} \zeta^{3} } \right)\,, $$

where

$$ \alpha_{1} = \frac{{G\,Log\left( {r_{1} /r_{2} } \right)}}{{R\tilde{L}\,Log\left( {r_{2} /R} \right)\,Log\left( {r_{1} /R} \right)}},\,\,\,\,\,\,\,\,\,\alpha_{2} = \frac{1}{R}\left( { - \frac{3}{2} + \frac{{Log\left( {r_{1} /R} \right) + Log\left( {r_{2} /R} \right)}}{{Log\left( {r_{1} /R} \right)\,Log\left( {r_{2} /R} \right)}}} \right), $$

and

$$ \alpha_{3} = \frac{1}{{R^{2} }}\left( {\frac{11}{6} - \frac{{2Log\left( {R^{2} /r_{1} r{}_{2}} \right)}}{{Log\left( {r{}_{2}/R} \right)Log\left( {R/r_{1} } \right)}} + \frac{{Log^{3} \left( {r{}_{2}/R} \right) + Log^{3} \left( {R/r_{1} } \right)}}{{Log\left( {r{}_{2}/r_{1} } \right)\left( {Log\left( {r{}_{2}/R} \right)Log\left( {R/r_{1} } \right)} \right)^{2} }}} \right). $$

Substituting Eqs. (12) and (15) into Eqs. (17–19) and (24), the solutions which are reliable with the preceding nonlinear border requirements may be formulated in the form:

25 $$ \phi_{j} = \frac{{ - h(r,r_{j} )\left( {\alpha_{1} \left( {\zeta + \alpha_{2} \zeta^{2} + \alpha_{3} \zeta^{3} } \right) + \rho_{j} \left( {\zeta_{t} + U_{0j} \zeta_{z} } \right)} \right)}}{{k\rho_{j} \left( {g(R,r_{j} ) - i\zeta_{z} h(R,r_{j} )\,} \right)}},\,\,\,j = 1,2, $$

26 $$ \psi_{1} = \frac{{H_{0} (\varepsilon_{m}^{(2)} - \varepsilon_{m}^{(1)} )h(r,r_{1} )\zeta_{z} \left( {h(R,r_{2} ) + i\,g(r_{2} ,R)\zeta_{z} } \right)}}{\Lambda }, $$

and

27 $$ \psi_{2} = \frac{{H_{0} (\varepsilon_{m}^{(2)} - \varepsilon_{m}^{(1)} )h(r,r_{2} )\zeta_{z} \left( {h(R,r_{1} ) + i\,g(r_{1} ,R)\zeta_{z} } \right)}}{\Lambda }, $$

where the functions $h(r,r_{j} ),\,g(r_{j} ,R)$ and $\Lambda$ are all listed in the Appendix.

(vi) To scrutinize the stability of the structure, the normal component of the stress tensor produces
  28 $$ \left\| {\rho \left( {\nabla \phi \cdot \nabla S} \right)\left( {\frac{\partial S}{{\partial t}} + \nabla \phi \cdot \nabla S} \right)} \right\| + \left( {\left\| P \right\| - \frac{1}{2}\left\| {\varepsilon_{m} \left( {H_{n}^{2} - H_{t}^{2} } \right)} \right\| + T\nabla \cdot n} \right)\left| {\nabla S} \right|^{2} = 0. $$

By removing the pressure in view of the equation of Bernoulli and substituting from the foregoing outcomes in Eq. (28), one may rewrite Eq. (28) as a nonlinear relationship in the following form:

29 $$ A_{1} \,\zeta_{tt} + T\zeta_{zz} + A_{2} \,\zeta_{zt} + A_{3} \,\zeta_{t} + A_{4} \,\zeta_{z} + A_{5} \,\zeta = N(\zeta ), $$

where $N(\zeta )$ denotes all nonlinear terms in $\zeta$. Additionally, $A_{1} ,\,A_{2} ,\,A_{3} ,\,A_{4}$ ${\text{and A}}_{{5}}$ are constants. To follow the text easily, they are moved to the Appendix.

## Linear stability analysis

At this stage, we shall scrutinize the linear stability methodology, while the nonlinear powers of the surface elevation $\zeta$ are neglected in Eq. (29). Subsequently, the linear dispersion equation can be formulated as:

30 $$ A_{1} \,\zeta_{tt} + T\zeta_{zz} + A_{2} \,\zeta_{zt} + A_{3} \,\zeta_{t} + A_{4} \,\zeta_{z} + A_{5} \,\zeta = 0. $$

A wave traveling formulae of Eq. (30) can be assumed as follows:

31 $$ \zeta (z,t) = \gamma \,e^{{i\left( {kz - \omega t} \right)}} + c.c. $$

Substituting Eq. (31) into (30), we achieve the dispersion relationship:

32 $$ a_{0} \omega^{2} + \left( {a_{1} + ib_{1} } \right)\omega + \left( {a_{2} + ib_{2} } \right) = 0, $$

where the coefficients $a_{0} ,\,a_{1} ,\,\,b_{1} ,\,\,a_{2} \,{\text{and}}\,b_{2}$ are listed in the Appendix.

The two cylinders with the central axis in Eq. (32) indicate the linear dispersion expression. The wave numeral and the development rate that is related to restrictions make up this amount. According to the linear stability strategy, the growth rate behavior determines if a history of time is stable or unstable. The instability will ultimately increase if the imaginary part is positive. Therefore, linear instability occurs in the mechanism. When the imaginary component is negative, the mechanism would be linearly stable since the disturbance would be reduced. Consequently, the stability or instability depends on how the mechanism of the actual component acts and whether it oscillates for any length of time. The primary objective of this section is to examine mechanical stability using a linear methodology. Employing the Routh-Hurwitz criteria^39^ to Eq. (32), we get the condition for stability (imaginary part $\omega_{I}$ of $\omega$ less than zero) as

33 $$ b_{1} > 0\quad {\text{and}}\quad {\text{a}}_{{1}} b_{1} b_{2} - a_{0} b_{2}^{2} - a_{2} b_{1}^{2} \ge 0. $$

After an uncomplicated computation, the second inequality may be written in a polynomial of $H_{0}^{2}$ in the following:

34 $$ \Gamma H_{0}^{2} + \tilde{\Gamma } > 0. $$

The coefficient**s** $\Gamma$ and $\tilde{\Gamma }$ are omitted to decrease the length of the article.

Henceforward, the stability conditions should be expressed in a dimensionless form, and the dimensionless amounts may be presented as:

$$ \begin{gathered} \omega^{*} = \omega \sqrt {\frac{{\rho_{1} R^{3} }}{T}} ,\,\,Oh = \frac{{\mu_{1} }}{{\sqrt {\rho_{1} TR} }},\,\,\,We = \frac{{\rho_{1} U_{01}^{2} R}}{T},\,\,\sigma = \frac{{\mu_{1} }}{{k_{1} R^{2} \rho_{1} }},\,\,\,Da = \frac{{k_{1} }}{{R^{2} }},\,\,\,\,L = \frac{{N_{1} }}{{\rho_{1} }},\,\,\alpha_{1}^{*} = \alpha_{1} \sqrt {\frac{{R^{3} }}{{\rho_{1} T}}} ,\,\,H_{0}^{*2} = \frac{{\varepsilon_{1} RH_{0}^{2} }}{T},\,\, \hfill \\ \varepsilon^{*} = \frac{{\varepsilon_{2} }}{{\varepsilon_{1} }},\,\,r_{j}^{*} = \frac{{r_{j} }}{R},\,\,N^{*} = \frac{{N_{2} }}{{N_{1} }},\,\,K^{*} = \frac{{K_{2} }}{{K_{1} }},\,\,\mu^{*} = \frac{{\mu_{2} }}{{\mu_{1} }},\,\,\rho^{*} = \frac{{\rho_{2} }}{{\rho_{1} }},\,\,U_{0}^{*} = \frac{{U_{02} }}{{U_{01} }},\,\,\nu_{j}^{\prime *} = \frac{{\nu^{\prime}_{j} }}{{R^{2} }},\,\,k^{*} = kR. \hfill \\ \end{gathered} $$

The star will be dropped for convenience.

Currently, to find out the influence of numerous physical factors involved in the system under consideration, we draw the variation $Log \, H_{0}^{2}$ against the wave number $k$ in Fig. $2 - 9$ by using Mathematica software, for the following parametric values:

$\rho = 3, \, \,{\text{Oh}} = {0}{\text{.6,}}\,\,{\text{We}} = {100,}\,\,L = 0.2,\,\,Da = 10,\,\mu = 0.6,\,\sigma = 5,\,\,N = 0.4,\,\,\nu^{\prime}_{1} \,\, = 0.3,\,\,\,\nu^{\prime}_{2} \,\, = 0.4,\,{\kern 1pt} \,\varepsilon_{m} = 0.2,\,\,\alpha_{1} = 0.4,\,\,U_{0} = 2.5$. The transition curve splits the diagram into a stable zone (S) above the curve, and an unstable zone (U) below the curve.

**Figure 2:**
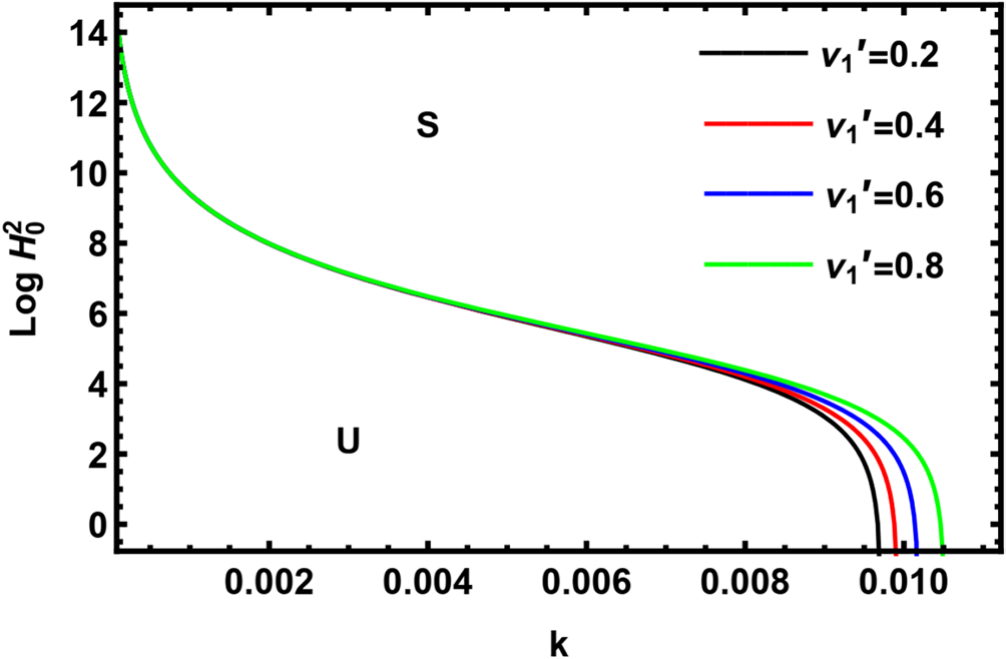
Shows the impact of the parameter $\nu_{1}^{^{\prime}}$.

**Figure 3:**
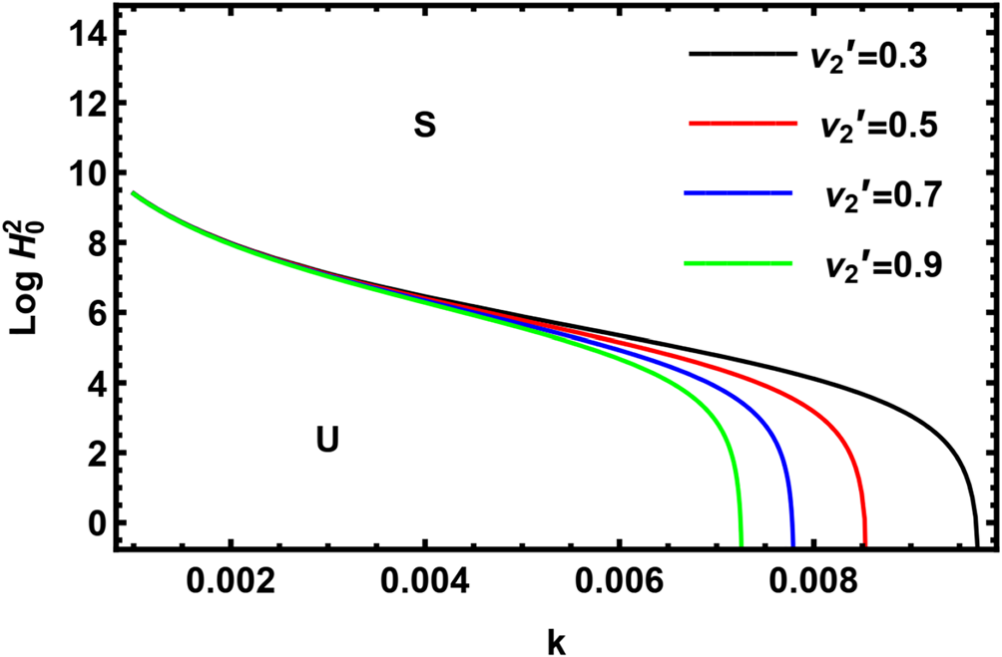
Demonstrates the impact of the parameter $\nu_{2}^{^{\prime}}$.

Figures 2 and 3 display the variation of $Log \, H_{0}^{2}$ with wave numeral $k$ for various amounts of the kinematic viscoelasticity of the pure inner fluid $\nu_{1}^{^{\prime}}$ and the kinematic viscoelasticity of the pure outer fluid $\nu_{2}^{^{\prime}}$. It is seen from Fig. 3 that the stability zone rises by increasing the kinematic viscoelasticity of the pure outer fluid. Hence, we conclude that the kinematic viscoelasticity of the pure outer fluid has a stabilizing influence on the considered structure. A similar outcome has been achieved by El-Sayed et al.^37^ and Awasthi et al.^40^. As viscoelasticity is existent in Eq. (1) with a negative sign, the energy dissipation will be less in the case of increasing elasticity. As less energy is obtained by the interface, the amplitude of the perturbation will be low, and disturbance will take more time to travel. i.e., the disturbance travels more slowly than usual. By contrast, Fig. 2 showed a destabilizing influence of the kinematic viscoelasticity of the pure inner fluid $\nu_{1}^{^{\prime}}$.

**Figure 4:**
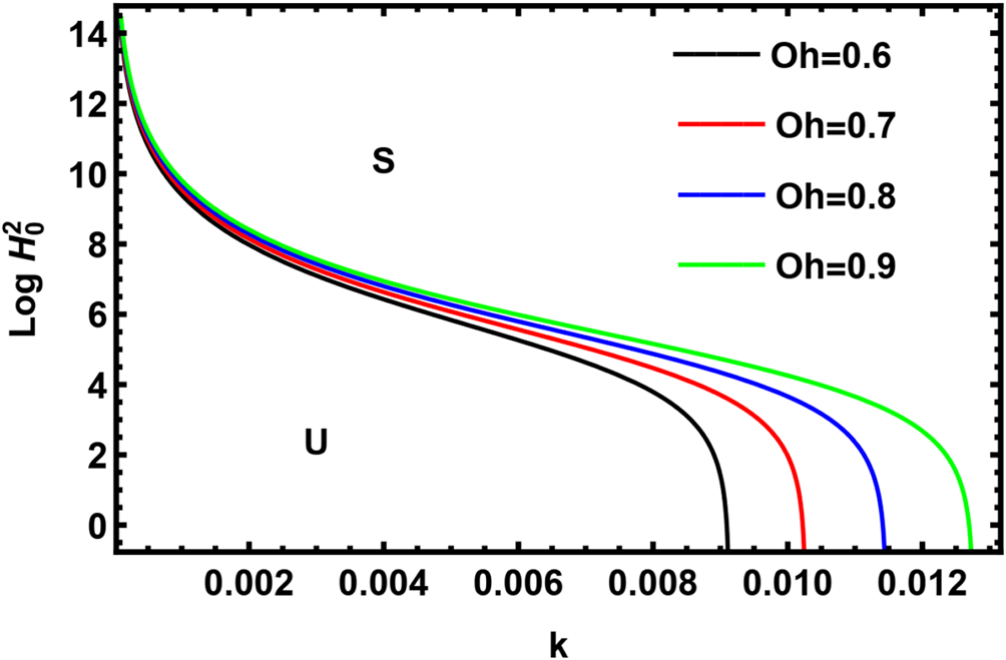
Displays the effect of the parameter $Oh$.

The variation of $Log \, H_{0}^{2}$ versus wave number $k$ has been plotted in Fig. 4 for different amounts of Ohnesorge numeral $Oh$. It is evident that the stability region decreases by increasing $Oh$. Hence, $Oh$ has a destabilizing influence; these results are compatible with recent studies by Moatimid et al.^41^. Through decreasing $Oh$, the density of the inside fluid and the surface tension increases and consequently the stability region increases. This concludes that the density of the inside fluid and the surface tension has a stabilizing nature.

**Figure 5:**
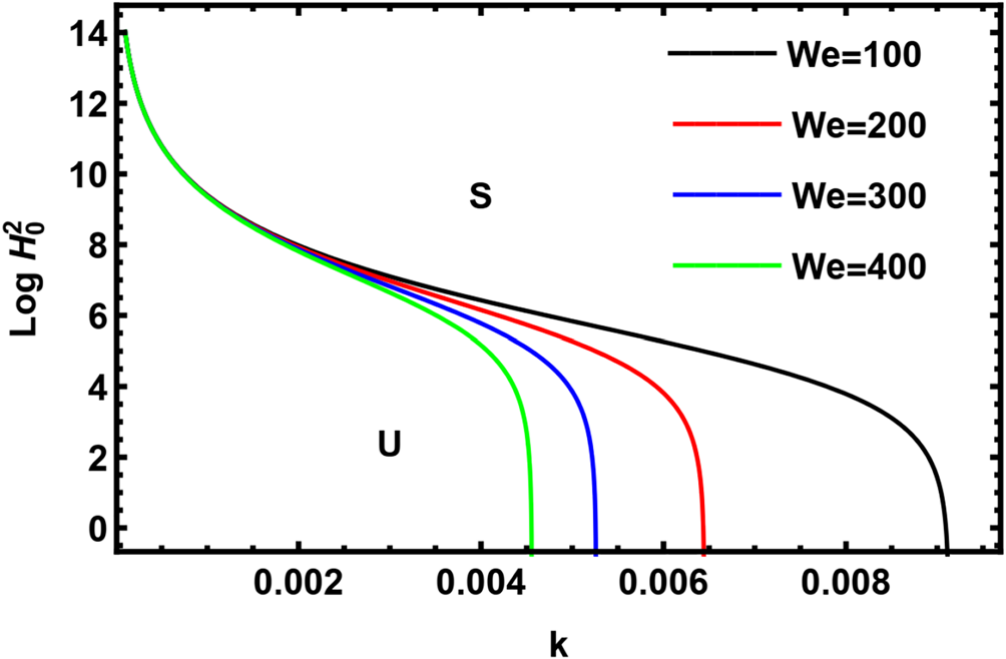
Displays the impact of the parameter $We$.

In Fig. 5, the transition curves of $Log \, H_{0}^{2}$ have been plotted for various amounts of Weber numeral $We$. The $We$ is the ratio of the inertial force to the surface tension force, which is helpful in examining liquid flows where there is a surface connecting two distinct liquids. It has been noticed that the stable zone rises by increasing the Weber numeral $We$, hence Weber number has a stabilizing effect. Therefore, one can conclude that the inertial force has a stabilizing effect on the interface while surface tension has a destabilizing nature. Our result agrees with Moatimid et al.^42^ and Awasthi et al.^40^.

**Figure 6:**
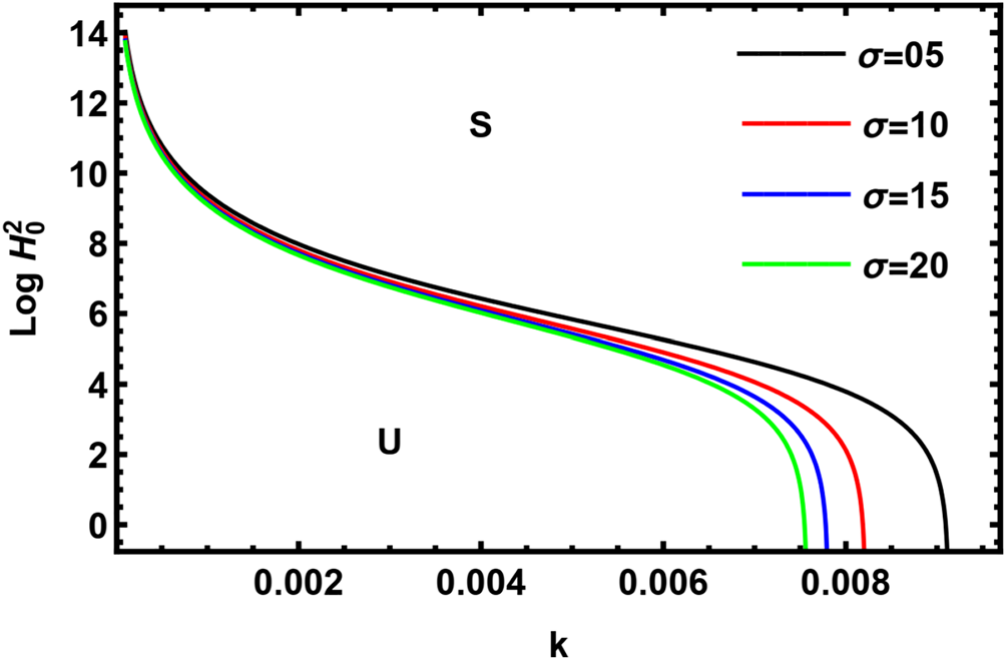
Shows the impact of the parameter $\sigma$.

The effect of the relaxation time parameter of dusty $\sigma$ on the neutral curves for $Log \, H_{0}^{2}$ is shown in Fig. 6. It is shown that $\sigma$ has a stabilizing impact on the considered scheme. Our result agrees with He et al.^43^. The relaxation time parameter of dusty $\sigma$ depends inversely on the Stocks drag coefficient. Hence, the Stocks drag coefficient destabilizes the interface. Compatible results were yielded in Ref.^43^.

**Figure 7:**
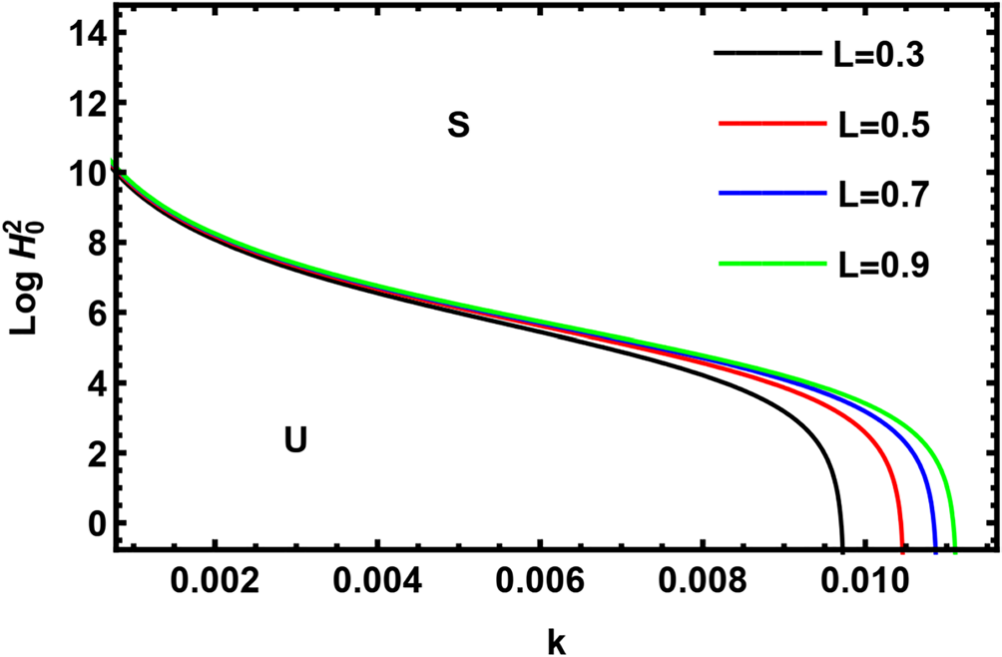
Shows the effect of parameter $L$.

The variation of neutral curves of $Log \, H_{0}^{2}$ against the wave number $k$ has been plotted in Fig. 7 for different values of mass concentration in dusty particles $L = 0.2,0.4\,\,{\text{ and 0}}{.6}$. It is observed that on increasing mass concentration in dusty particles, the stability region decreases, hence $L$ has a destabilizing effect. Our result agreed with He et al.^43^. The mass concentration in dusty particles depends directly on the density number of the suspended inner dust particles $N_{1} \,$ and inversely on the density of the pure inner fluid $\rho_{1}$. Hence, $N_{1} \,$ shows a destabilizing effect, while $\rho_{1}$ stabilizes the interface. The same results are obtained from Figs. 4 and 5. Therefore, $\rho_{1}$ has a stabilizing nature.

**Figure 8:**
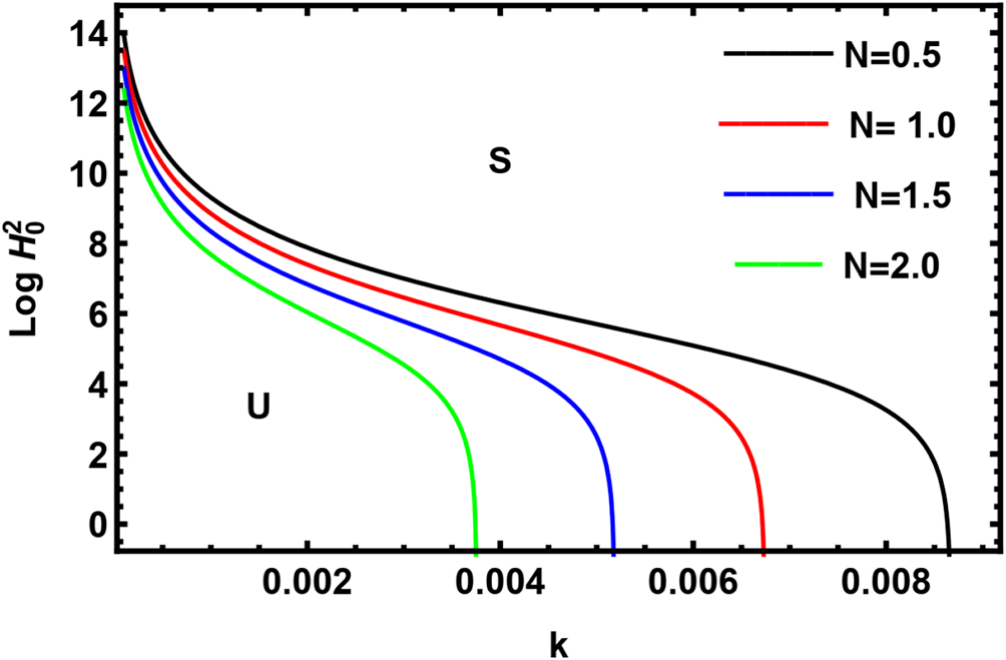
Displays the impact of the parameter $N$.

Figure 8 shows the neutral curves for $Log \, H_{0}^{2}$ in light of different values of the density number ratio of the suspended dust particles $N$. It is noticed that the unstable zone is maximum when the numeral density of the suspended inner dust particles is equal to the density number of the suspended outer dust particles. If the density number of the suspended inner dust particles is larger than the density number of the suspended outer dust particles, the stability range decreases, while the interface will be more stable if the density number of the suspended outer dust particles is larger than the density number of the suspended inner dust particle. Consequently, it is concluded that the density number of the suspended outer dust particles has a stabilizing nature, and the numeral density of the suspended inner dust particles has a destabilizing impact on the given structure. A similar outcome has been achieved from Fig. 7.

**Figure 9:**
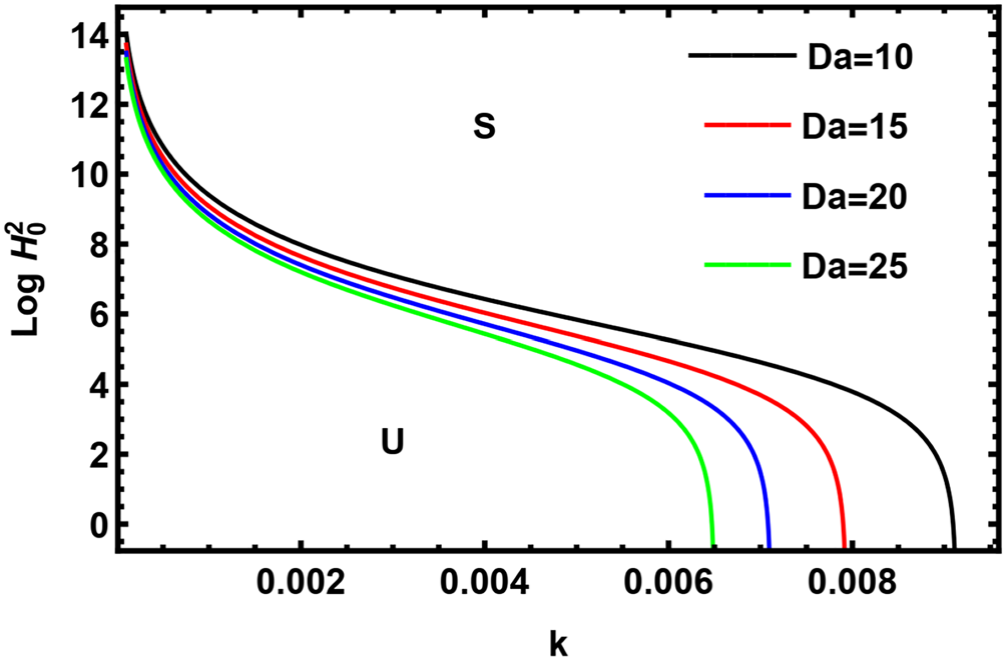
Displays the impact of the parameter $Da$.

Figure 9 displays the deviation of $Log \, H_{0}^{2}$ with regard to wave number $k$ for different amounts of Darcy numeral (permeability parameter). The increase in the Darcy numeral tends to stabilize the present system. Darcy numeral depends directly on permeability of the medium. Hence, permeability of the medium shows a destabilizing effect. The stabilizing impact is in accord with Ref.^42^.

**Figure 10:**
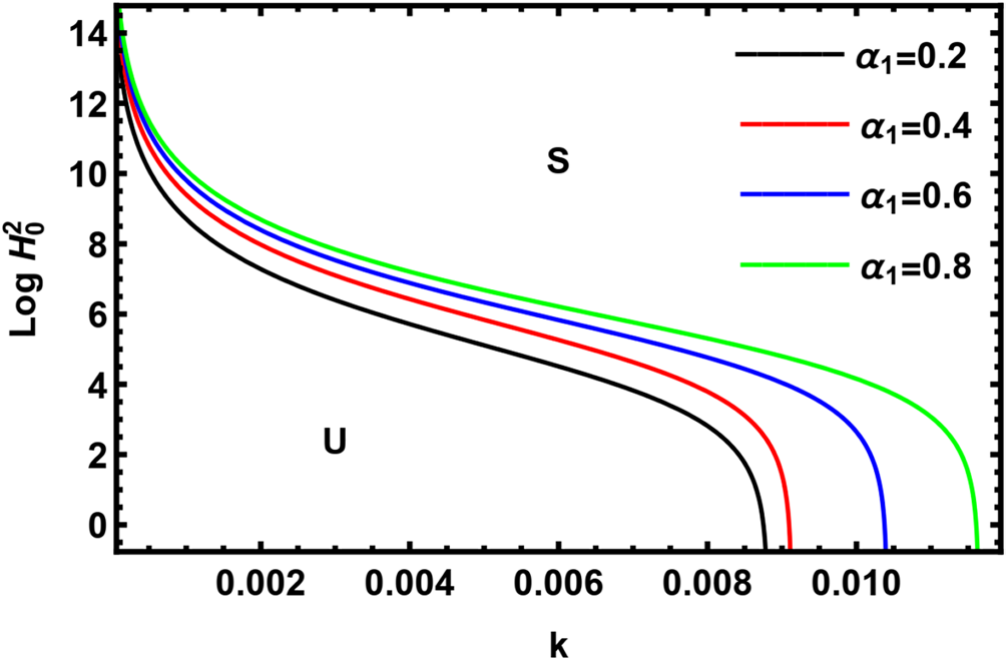
Shows the impact of the parameter $\alpha {}_{1}$.

Figure 10 depicts the influence of the coefficient of heat transfer $\alpha {}_{1}$, which is the ratio of net temperature flux to the latent heat on the neutral curves for $Log \, H_{0}^{2}$. It is found that $\alpha {}_{1}$ has a slightly destabilizing influence on the stability picture of the considered structure. This result agreed with the previous studies^44,45^.

**Figure 11:**
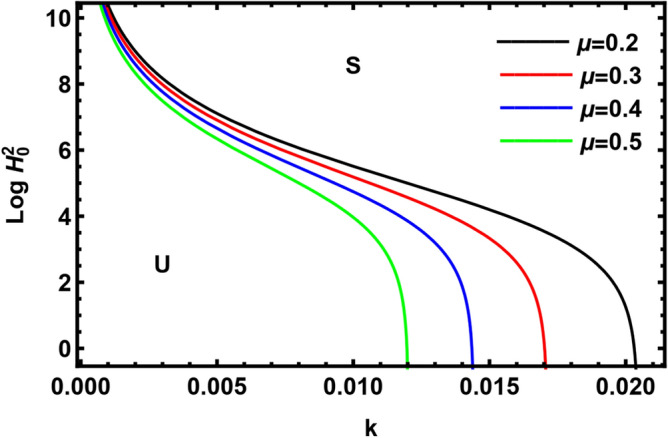
Displays the parameter $\mu$.

Figure 11 displays the deviation of $Log \, H_{0}^{2}$ with regard to the wave numeral $k$ for various amounts of the viscosity ratio $\left( {\mu = 0.2,\,0.3,\,\,0.4\,\,{\text{and}}\,0.5} \right)$. It is shown that the stable zone rises as $\mu$ raises. So, it is concluded that the viscosity ratio has a stabilizing influence. This outcome agrees with that of El-Sayed et al.^37^ and Awasthi et al.^40^. As well-known, the viscosity ratio $\mu$ is directly proportional to viscosity of the pure outer liquid and inversely proportional to the viscosity of the pure inner liquid. Consequently, the viscosity of the pure outer fluid has a stabilizing impact, while the viscosity of the pure inner liquid plays a destabilizing role.

## Nonlinear stability analysis

The purpose of what follows is to investigate the nonlinear stability of the considered system. As presented within the analysis of linear stability, the displacement of the perturbed surface $\zeta (z,t)$ has a distinctive shape that is given in Eq. (31). The nonlinear Eq. (29) can be rewritten in the following algebraic equation:

35 $$ D(\omega ,k)\zeta = \lambda_{1} (\omega ,k)\zeta^{2} + \lambda_{2} (\omega ,k)\zeta^{3} , $$

where $D(\omega ,k),\,\,\lambda_{1} (\omega ,k)$ and $\,\lambda_{2} (\omega ,k)$ indicate the linear and nonlinear coefficients, respectively. These terms are lengthy, so they will not be included here.

The procedure for examining the stability analysis of Eq. (35) has been discussed in detail by several previous works as in Refs.^10,11,42^. Consequently, applying an analogous point of view as shown in the previous literature, the following Ginzburg–Landau equation is found as follows:

36 $$ i\frac{\partial \gamma }{{\partial \tau }} + P\frac{{\partial^{2} \gamma }}{{\partial \eta^{2} }} = Q\gamma^{2} \overline{\gamma } , $$

where

$$ P = \left( {P_{i} + P_{r} } \right) = - \frac{1}{2}\left( {V_{g}^{2} \frac{{\partial^{2} D}}{{\partial \omega^{2} }} + 2V_{g} \frac{{\partial^{2} D}}{\partial \omega \,\partial k} + \frac{{\partial^{2} D}}{{\partial k^{2} }}} \right)\left( {\frac{\partial D}{{\partial \omega }}} \right)^{ - 1} , $$

$$ Q = Q_{i} + Q_{r} = \left( {\frac{{2\lambda_{1} }}{\Omega } + 3\lambda_{2} } \right)\,\left( {\frac{\partial D}{{\partial \omega }}} \right)^{ - 1} , $$

$$ V_{g} = - \frac{\partial D}{{\partial \,k}}\left( {\frac{\partial D}{{\partial \omega }}} \right)^{ - 1} , $$

$$ \Omega = D\left( {2\omega ,2k} \right), $$

37 $$ \eta = \delta (z - V_{g} t),\,\,\,\,\,\,\,\,\,{\text{and}}\,\,\,\,\,\,\,\,\,\,\,\,\tau = \delta^{2} t, $$

Here, $V_{g}$ indicates the group velocity.$P$ and $Q$ are lengthy terms, so they are not included here. Upon request, they are available with the authors.

The stability analysis of the Ginzburg–Landau Eq. (36) has been previously examined^46^. It follows that the structure is stable in accordance with:

38 $$ Q_{i} < 0,\,\,\,\,\,\,\,\,\,{\text{and}}\,\,\,\,\,\,\,\,P_{r} Q_{r} + P_{i} Q_{i} > 0. $$

If not, the system is unstable. The transition curves separating the stable from the unstable zone correspond to:

39 $$ \,P_{r} Q_{r} + P_{i} Q_{i} = 0, $$

and

40 $$ Q_{i} = 0. $$

It is convenient for a nonlinear numerical discussion to write the conditions of stability in non-dimensional quantities. Therefore, imagine the following non-dimensional forms: $\left( h \right)$, $\left( {1/\omega } \right)$ and $T/\omega^{2}$ refer to length, time, and mass, correspondingly. After lengthy, but direct calculations, the transition curve $P_{r} Q_{r} + P_{i} Q_{i} = 0$ may be written in a fifth-degree polynomial on $H_{0}^{2}$ as:

41 $$ L_{5} (H_{0}^{2} )^{5} + L_{4} (H_{0}^{2} )^{4} + L_{3} (H_{0}^{2} )^{3} + L_{2} (H_{0}^{2} )^{2} + L_{1} H_{0}^{2} + L_{0} = 0. $$

Meanwhile**,** the transition curve $Q_{i} = 0$ can be arranged a polynomial on $H_{0}^{2}$ as:

42 $$ Q_{3} (H_{0}^{2} )^{3} + Q_{2} (H_{0}^{2} )^{2} + Q_{1} H_{0}^{2} + Q_{0} = 0. $$

The coefficients of Eqs. (41) and (42) are very lengthy, so they will be omitted to prevent the size of the article.

**Figure 12:**
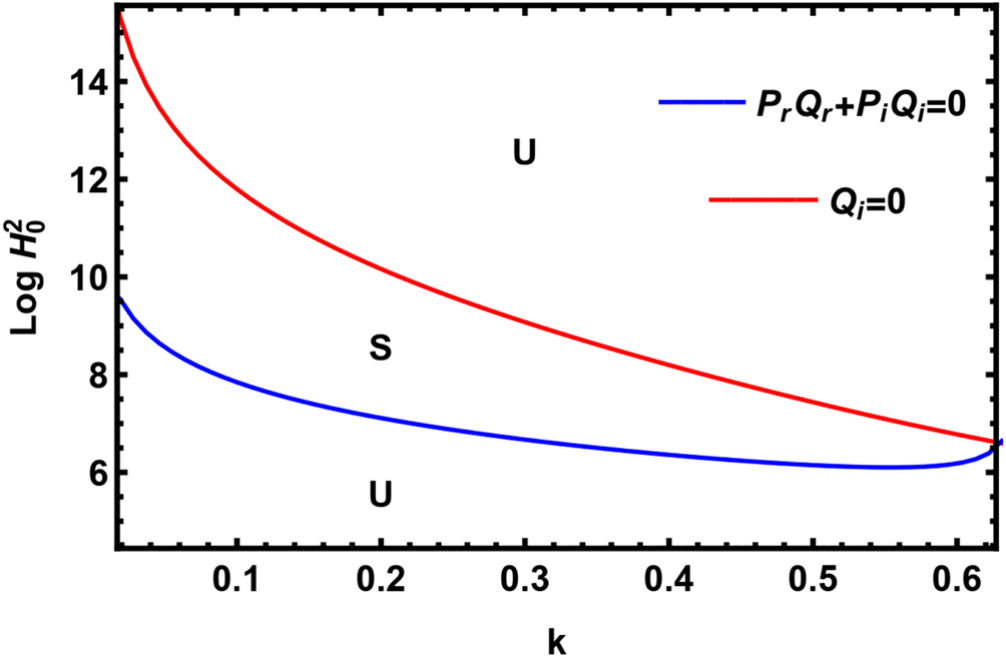
The natural curves, as presented in Eqs. (39) and (40).

It should be mentioned that several numeral curves do not appear in Fig. 12, because they are either negative or complex.

To clarify the stability standards through the nonlinear stability analysis, the numeral curves as presented in Eqs. (41) and (42) are designed in Fig. 12. As demonstrated in this diagram, the red curve signifies one positive real root from Eq. (42), meanwhile, the blue curve signifies one positive real root from Eq. (41), where the other roots from Eqs. (41) and (42) are either negative or complex. As seen from Fig. 12, the nonlinear stability zone is the region connecting the red and blue curves. The zones of stability/instability nonlinear analysis are symbolized by letters S and U. It is obvious from this diagram that magnetic field has a destabilizing impact. This result agrees with the earlier work^47^.

**Figure 13:**
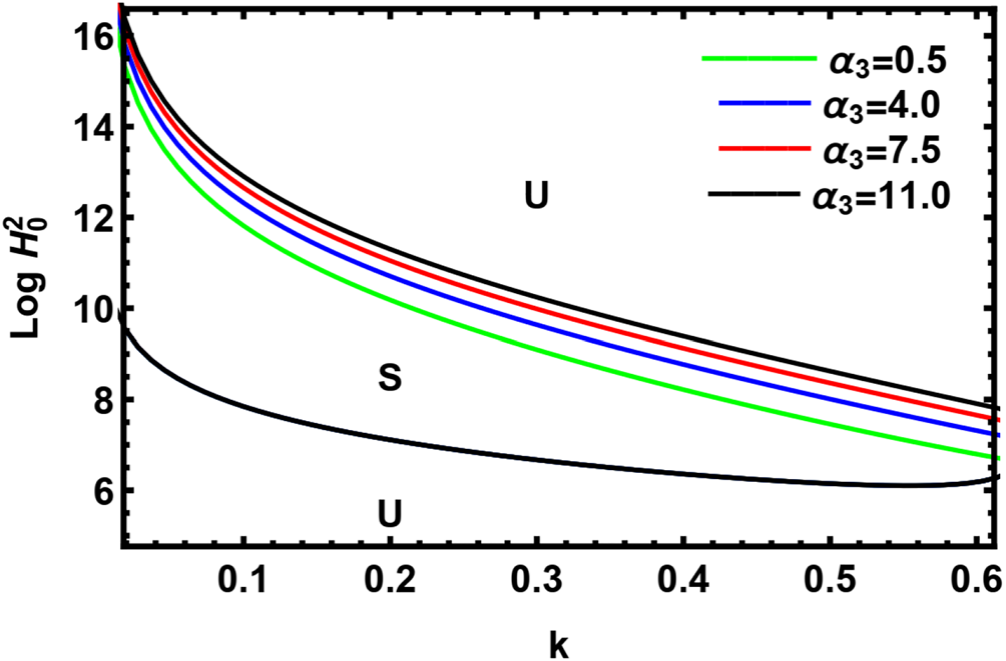
The transition curve with the variation $\alpha_{3}$.

In Fig. 13, we have displayed the impact of the MHT on the parameter $\alpha_{3}$. As realized from this diagram, the nonlinear stability region is regulated by a curve dependent only on a positive real root from Eq. (42). The figure shows that as $\alpha_{3}$ rises, the stability zone rises. Hence, the presence of a magnetic field doesn't influence the nature of heat transfer. A similar result was observed by Dharamendra and Awasthi^48^. This shows that MHT has a stabilizing influence. This outcome has been proven by several previous researchers; for instance, see Agarwal and Awasthi^49^.

**Figure 14:**
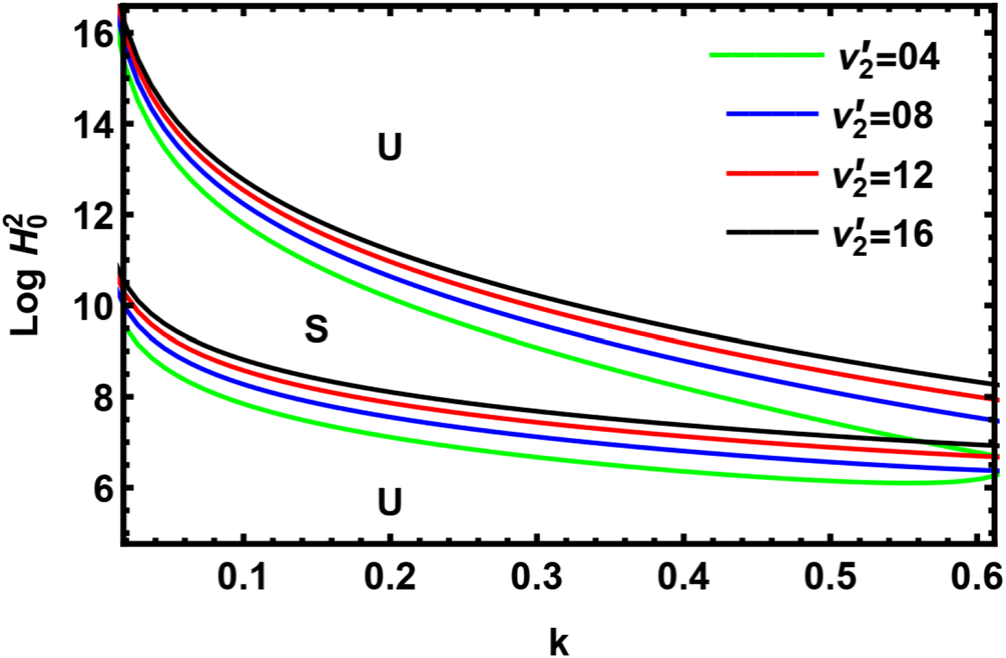
The neutral curve with the deviation of $\nu^{\prime}_{2}$.

The impact of the kinematic viscoelasticity of the pure outer liquid $\nu^{\prime}_{2}$ is examined through Fig. 14. It is noticed that the factor $\nu^{\prime}_{2}$ has a stabilizing effect. Comparable findings were found in Awasthi et al.^40^. This outcome is in good accord with the previous outcomes as shown in Fig. 3 through the linear theory approach. As known, when the viscoelasticity increases, the energy dissipation decreases because the interface gets less power. Therefore, the perturbation amplitude decreases, and the perturbation will take longer to travel. Consequently, the viscoelasticity has a stabilizing nature. This result agrees with the earlier work^37^.

**Figure 15:**
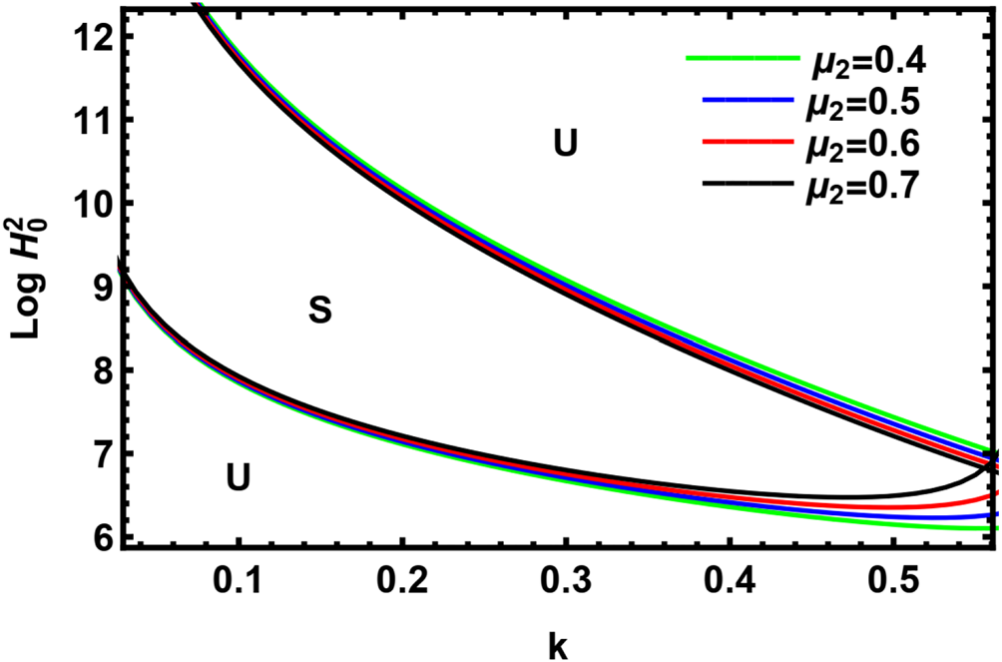
The neutral curve with the deviation of $\mu_{2}$.

In Fig. 15, the impact of viscosity of the pure outer liquid is depicted. It is noticed that the rise in the viscosity of the pure outer liquid decreases the stable region in the nonlinear analysis, and this shows that $\mu_{2}$ has a destabilizing impact. This outcome is in accord with that of El-Sayed and Al-Harbi^50^. Additionally, the viscosity in the viscous potential flow theory has a destabilizing impact^51^.

**Figure 16:**
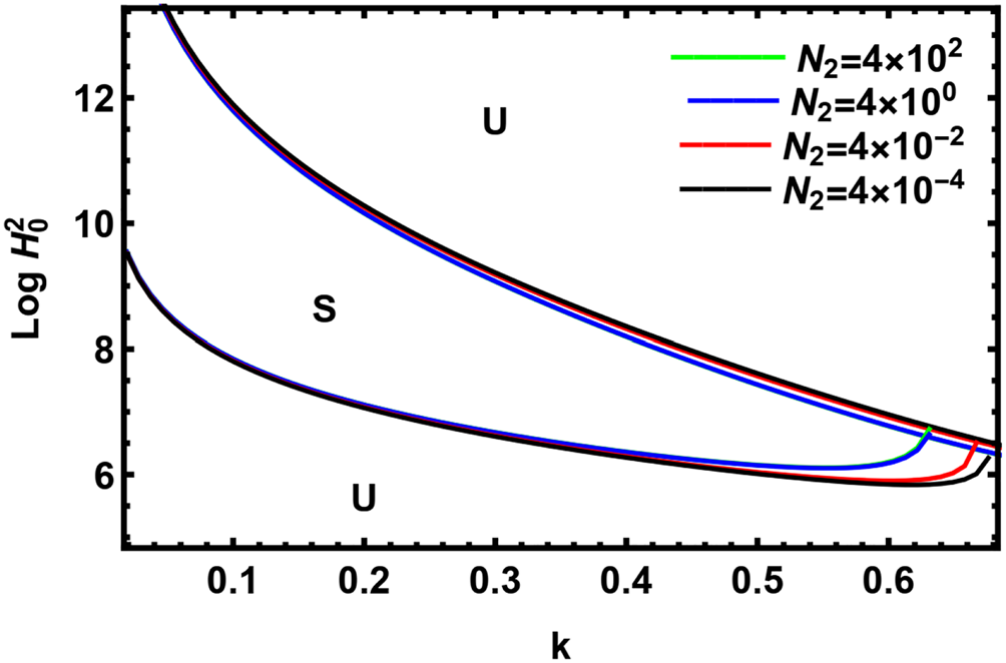
The transition curve with the variation $N_{2} \,$.

Figure 16 is devoted to indicating the effect of the density number of the suspended outer dust particles $N_{2} \,$, it is obvious from this figure that $N_{2} \,$ has slightly a stabilizing impact on the considered structure. This outcome coincides with the conclusion shown in Fig. 8.

## Approximate interface displacement profile

In this section, an analytic estimated solution for the perturbed interface is presented. A comparison between the numerical solution and the analytical solution of the perturbed interface is described. As previously obtained, we have obtained the nonlinear characteristic Eq. (29), which is represented by a nonlinear PDE of second order with complex coefficients of the perturbed interface $\zeta (z,t)$. To facilitate the subsequent calculations, we can assume that:

43 $$ \zeta (z,t) = u(t)\,\,e^{i\,k\,z} . $$

It will be easier to make the following calculations if only time dependence i.e., $\zeta = \zeta (0,t) = u(t)$ is considered, which is the true indicator of historical stability. Under this assumption, we can rewrite the nonlinear characteristic equation Eq. (29) as

44 $$ u^{\prime\prime}(t) + \,\frac{1}{{m_{1} }}\left( {m_{3} + i\left( {km_{2} + m_{4} } \right)} \right)u^{\prime}(t) + \frac{1}{{m_{1} }}\left( { - km_{6} + m_{7} - k^{2} m_{9} + i\left( {km_{5} + m_{8} } \right)} \right)u(t) + N_{1} (u^{2} ) + N_{2} (u^{3} ) = 0, $$

where $N_{1} (u^{2} )$ and $N_{2} (u^{3} )$ represents all nonlinear terms. The coefficients $\,m_{i} \,\,(i = 1,2,...9)$ are lengthy, so they will be omitted. They are available from the author on request.

Separating real and imaginary parts of Eq. (44), then eliminating the term $u^{\prime}\,(t)$, we get the following:

45 $$ u^{\prime\prime}(t) + \tilde{\omega }^{2} u(t) = \,\hbar \left( {Y_{0} \,u^{2} (t) + Y_{1} u(t)\,\,u^{\prime\prime}(t) + Y_{2} \,u^{3} (t) + Y_{3} \,u^{2} (t)\,\,u^{\prime\prime}(t)} \right)\,,\,\,\,\,\,\,\,\hbar \in [0,1], $$

where

$$ \tilde{\omega }^{2} = \frac{1}{{km_{1} \left( {m_{2} + m_{4} } \right)}}\left[ { - m_{3} m_{8} + m_{4} m_{7} - k\left( {m_{3} m_{5} + m_{4} m_{6} - m_{2} m_{7} } \right) - k^{2} \left( {m_{2} m_{6} + m_{4} m_{9} } \right) - k^{3} m_{2} m_{9} } \right]. $$

$\tilde{\omega }^{2}$ indicates the natural frequency of the present model, the coefficients $\,Y_{i} \,\,(i = 0,1,2,3)$ are recognized from the background, and $\hbar$ is known as the homotopy parameter.

Equation (45) represents a generalized cubic nonlinear differential equation; to solve this equation, the following primary requirements can be proposed:

46 $$ u(0) = 0\quad {\text{and}}\quad u^{\prime}(0) = 1. $$

To get an analytical estimate solution of Eq. (45), the evaluation will be established on the sequence of HPM with Laplace transforms. This approach will be based on the expansion of the nonlinear frequency $\varpi^{2}$ to be stated as in Ref.^11^:

47 $$ \varpi^{2} = \tilde{\omega }^{2} + \sum\limits_{j = 1}^{\infty } {\hbar^{j} \,\Re_{j} } . $$

In accordance with the HPM, the expanded form of the time-dependent function $u(t;\hbar )$ can be formulated as:

48 $$ u(t;\hbar ) = \sum\limits_{j = 0}^{\infty } {\hbar^{j} \,\,u_{j} \,(t)\,} = u_{0} \,(t) + \,\hbar u_{1} \,(t) + \hbar^{2} u_{2} \,(t) + \cdots $$

By substituting Eq. (47) in Eq. (45), the following nonlinear characteristic equation is obtained:

49 $$ u^{\prime\prime}(t) + \varpi^{2} u(t) = \,\hbar \left( {Y_{0} \,u^{2} (t) + Y_{1} u(t)\,\,u^{\prime\prime}(t) + Y_{2} \,u^{3} (t) + Y_{3} \,u^{2} (t)\,\,u^{\prime\prime}(t) + (\Re_{1} + \,\hbar \,\Re {}_{2}\, + \hbar^{2} \,\Re_{3} )\,\,u(t)} \right). $$

By computing the Laplace transform to Eq. (49) and considering the preliminary requirements specified in Eq. (46), the following equation is obtained:

50 $$ L_{T} \{ \,u(t;\hbar )\} = \frac{1}{{S^{2} + \varpi^{2} \,}} + \,\frac{\hbar \,\,}{{s^{2} + \varpi^{2} \,}}L_{T} \left( {\,Y_{0} \,u^{2} (t) + Y_{1} u(t)\,\,u^{\prime\prime}(t) + Y_{2} \,u^{3} (t) + Y_{3} \,u^{2} (t)\,\,u^{\prime\prime}(t) + (\Re_{1} + \,\hbar \,\Re {}_{2}\, + \hbar^{2} \,\Re_{3} )\,\,u(t)} \right)\,.\, $$

Taking the inverse Laplace transforms $L_{T}^{ - 1}$ of both sides of Eq. (50), we find:

51 $$ \,u(t;\hbar ) = \frac{1}{\varpi }\,\sin \,\varpi \,t + L_{T}^{ - 1} \,\left[ {\frac{\hbar \,}{{s^{2} + \varpi^{2} \,}}L_{T} \left( {\,Y_{0} \,u^{2} (t) + Y_{1} u(t)\,\,u^{\prime\prime}(t) + Y_{2} \,u^{3} (t) + Y_{3} \,u^{2} (t)\,\,u^{\prime\prime}(t) + (\Re_{1} + \,\hbar \,\Re {}_{2}\, + \hbar^{2} \,\Re_{3} )\,\,u(t)} \right)} \right]. $$

Substituting Eq. (48) in Eq. (51), and after comparing the quantities of identical power of $\hbar$ on equal sides, the following equations are obtained:

52 $$ \hbar^{0} :\,\,\,u_{0} \,(t) = \frac{1}{\varpi }\,\sin \,\varpi \,t\,, $$

53 $$ \hbar :\,u_{1} \,(t) = L_{T}^{ - 1} \,\left[ {\frac{1}{{s^{2} + \varpi^{2} \,}}L_{T} \left\{ {\Re_{1} \,u_{0} \,(t)} \right.\, + \,\,Y_{0} \,u_{0}^{2} (t) + Y_{1} \,u_{0} (t)\,u^{\prime\prime}_{0} (t)\, + \,Y_{2} \,u_{0}^{3} (t)\, + \,Y_{3} u_{0}^{2} (t)u^{\prime\prime}_{0} (t)} \right], $$

Substituting Eq. (52) into Eq. (53) and cancelling the secular terms, the coefficients of $\sin \,\,\varpi \,\,t$ and $\cos \,\,\varpi \,\,t$ will be eliminated. According to this procedure, one gets the values of ${\mathfrak{R}}_{1}$ as follows:

54 $$ \Re_{1} = \frac{3}{4}\left( {Y_{3} - \frac{{Y_{2} }}{{\varpi^{2} }}} \right). $$

Substituting Eq. (54) into Eq. (47), we get the following fourth order equation in the new nonlinear frequency:

55 $$ \varpi^{4} - (\omega^{2} + \frac{3}{4}Y_{3} )\,\,\varpi^{2} + \frac{3}{4}Y_{2} = 0. $$

This equation can be solved to get the value of $\varpi$ as follows:

56 $$ \varpi = \pm \frac{1}{2\sqrt 2 }\sqrt {4\omega^{2} + 3Y_{3} + \sqrt {(4\omega^{2} + 3Y_{3} )^{2} - 48Y_{2} } } . $$

Substituting Eqs. (54) and (52) into Eq. (53), we get the periodic solution of $\delta_{1} \,(t)$ as follows:

57 $$ u_{1} \,(t) = \frac{{\,(\,Y_{0} - \varpi^{2} Y_{1} )}}{{2\,\varpi^{4} \,}} + \frac{{\,3(\, - Y_{2} + \varpi^{2} Y_{3} )}}{{32\,\varpi^{5} \,}}\sin \,\,\,\varpi \,t + \,\frac{{\,2(\, - Y_{0} + \varpi^{2} Y_{1} )}}{{3\,\varpi^{4} \,}}\cos \,\,\varpi \,t + \frac{{\,(\,Y_{0} - \varpi^{2} Y_{1} )}}{{6\,\varpi^{4} \,}}\,\cos \,\,\,2\varpi \,t + \,\frac{{\,(\,Y_{2} - \varpi^{2} Y_{3} )}}{{32\,\varpi^{5} \,}}\,\sin \,3\varpi \,t\,\,. $$

Consequently, the bounded estimated solution of the time-dependent function $u(t)$ can be written as:

58 $$ u(t) = \lim_{h \to 1} (u_{0} + \hbar u_{1} + \cdots ), $$

where $u_{0}$ and $u_{1}$ are presented by Eqs. (52) and (57), correspondingly.

In this part of the investigation, a numerical calculation of the obtained theoretical result is carried out. The dimensionless quantities are very effective in simplifying the achieved outcomes. For this objective, one considers the same dimensionless quantities as provided in the non-linear approach. To establish the numerical results, the diagrams are designed for a structure receiving the following specifics:

$$ \begin{aligned} & \mu_{1} = 3,\,\,\mu_{2} = 1,\nu^{\prime}_{1} \,\, = 4,\,\,\,\nu^{\prime}_{2} \,\, = 1,\rho_{1} = 10,\,\,\rho_{2} = 1,\,\,U_{01} = 0.2,\,\,U_{02} = 1,\,\,\alpha_{1} = 0.1,\,\,\alpha_{2} = 0.3,\,\,\alpha_{3} = 0.5,\,\,r_{1} = 0.5,\,\,r_{2} = 0.9, \\ & K_{1} = 1,K_{2} = 20,\,N_{1} = 1,\,\,N_{1} = 5,\,\,\varepsilon_{1} = 1,\,\,\varepsilon_{2} = 10\,\,{\text{and }}\,{\text{H}}_{{0}} = 100. \\ \end{aligned} $$

**Figure 17:**
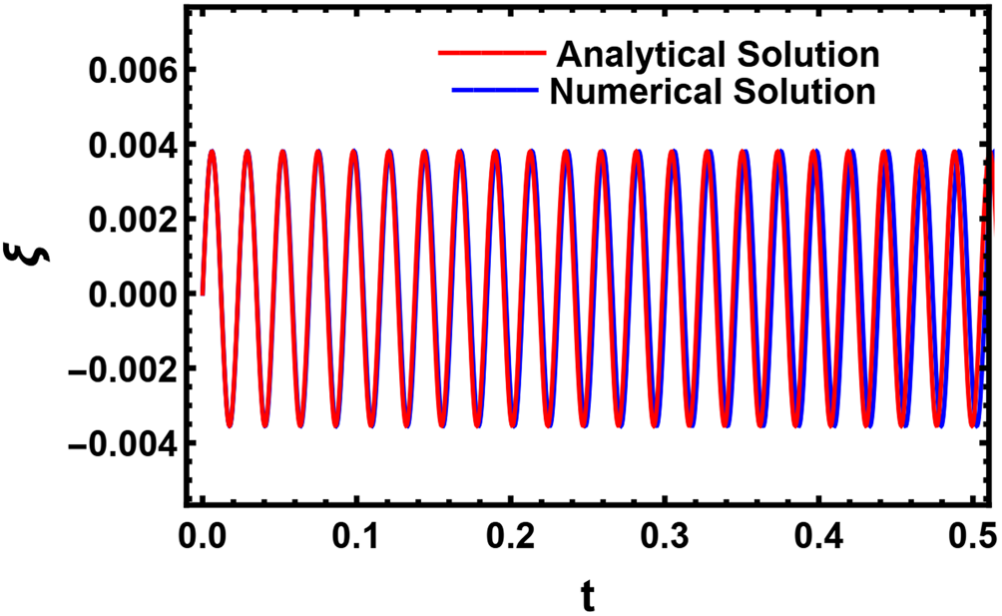
A comparison between the analytical and the numerical solutions of the perturbed interface.

Therefore, Fig. 17 displays the estimated solution as given in Eq. (58). As seen, the solution behaves as a uniform wave. This occurs owing to the cancellation of all secular terms. It should be noted that the traditional HPM resulted in a growth rate solution which is physically undesired. Therefore, in accordance with the modification of the HPM, the concept of the nonlinear expanded frequency enables us to avoid the sources of these secular terms. Simultaneously, this figure represents a comparison between the analytical solution of the perturbed interface as given in Eq. (45) and a numerical methodology to validate the theoretical outcome. It is realized that the two solutions are consistent.

## Concluding remarks

We have presented a detailed analysis of the linear and nonlinear stability of a vertical cylindrical perturbed surface between two dusty Rivlin-Ericksen magnetic fluids, with MHT across the interface. Axisymmetric disturbance is considered throughout this study. The work finds its significance in wastewater treatment and petroleum transport. The linear fundamental formulae with the applicable nonlinear border restrictions have developed in a characteristic nonlinear PDE that governs the interface displacement. Through the Routh-Hurwitz theory, linear stability standards have been achieved. The method of multiple scales has been employed, where the nonlinear Ginzburg–Landau equation is achieved. This equation governs the nonlinear stability benchmarks of the considered system. Therefore, the stability condition of nonlinear analysis is achieved and examined both analytically and numerically. A set of diagrams is displayed to indicate the impacts of several physical factors. Finally, the HPM with the conception of nonlinear expanded frequency is used to achieve an estimated solution for the perturbed interface. A good quality matching is achieved when compared to the numerical solution. The final outcomes of the whole study may be summed up as follows:

- Along with the linear analysis, one gets:
- The structure is stable when the numeral density of the suspended inner dust particles is less than the density number of the suspended outer dust particles, and vice-versa.
- The relaxation time factor of dust particles, Weber number and Darcy number have a stabilizing influence on the studied construction.
- The Ohnesorge number, mass concentration in dust particles and the HMT have destabilizing effects on the construction.
- The structure is stable if the kinematic viscoelasticity of the pure outer fluid is greater than the kinematic viscoelasticity of the inner liquid.
- The density of the pure inner liquid and the surface tension has a stabilizing influence on the structure.
- The obtained results in the nonlinear analysis can be outlined as follows:
- The stability of the structure increases via decreasing the magnetic strength.
- The increase of the nonlinear MHT enhances the stability of the scheme.
- The kinematic viscoelasticity of the pure outer liquid and the numeral density of the suspended outer dust particles have a stabilizing influence on the stability of the structure. These results are the same as those achieved in the linear study.
- The stable range decreases with an enhancement in the viscosity of the pure outer liquid, that is, the viscosity of the pure outer fluid has a destabilizing effect.
- In light of the HPM, with the help of the frequency configuration, a uniform estimate solution of the surface displacement is achieved. This solution is in good agreement and is validated via RK4.

### Further recommendations

1. The existence of the energy and the concentration equations will be provided together away from with Hsieh's simplification.
2. Different nanofluids with the several non-Newtonian fluid types will be adopted.
3. In light of the significance of dusty fluids in diverse practical engineering applications, various stability problems in plane geometry will be handled.
4. Time-varying external fields will be supplied.
5. A non-perturbative advanced method can be used to examine the stability methodology in light of the non-perturbative approaches in evaluating the nonlinear PDEs.

## Supplementary Information

Supplementary Information.

## Data Availability

All data generated or analyzed during this study are included in this manuscript.

## English symbols

$$P$$: Hydrostatic pressure $$({\text{kg}}\,{\text{m}}^{ - 1} \,{\text{s}}^{ - 2} )$$
$N_{1} \,$: Density number of suspended inner dust particles
$N_{2} \,$: Density number of suspended outer dust particles
$H_{0}$: Uniform axial magnetic field (Tesla)
$K = 6\pi \mu \eta$: Coefficient of Stokes drags
$$k$$: Wave number ($${\text{m}}^{ - 1}$$)
$\underline{U} (r,z;t)$: Velocity of pure liquid $({\text{m}}\,{\text{s}}^{ - 1} )$
$\underline{V}$: Velocity of the dust particles $({\text{m}}\,{\text{s}}^{ - 1} )$
$T_{0}$: Temperature at the interface $({\text{Kelvin}})$
$T_{1}$: Temperature in the inner cylinder $({\text{Kelvin}})$
$T_{2}$: Temperature in the outer cylinder $({\text{Kelvin}})$
$r_{1}$: Radius of the inner rigid cylinder $({\text{m}})$
$r_{2}$: Radius of the outer rigid cylinder $({\text{m}})$
$\underline{e}_{r}$: Unit vectors along $r{ - }$direction
$\underline{e}_{z}$: Unit vectors along $z{ - }$direction
$I_{0} (kr)$: Modified Bessel function of the first kind of order zero
$K_{0} (kr)$: Modified Bessel function of the second kind of order zero
$$k_{1}$$: Medium permeability $$({\text{m}}^{2} )$$
Oh: Ohnesorge number
We: Weber number
Da: Darcy number
$L$: Mass concentration in dust particles
$\tilde{L}$: The latent heat released during phase transformation
T: Surface tension $$({\text{dyn}}\,{\text{cm}}^{ - 1} )$$
$c.c.$: Complex conjugate of the preceding term
$\rho_{1} \,$: Density of pure inner fluid $({\text{kg}}\,{\text{m}}^{ - 3} )$
$\rho_{2} \,$: Density of pure outer fluid $({\text{kg}}\,{\text{m}}^{ - 3} )$
$\mu^{\prime}_{1} \,\,\,$: Viscoelasticity of pure inner fluid $({\text{kg}}\,{\text{m}}^{ - 1} )$
$\mu^{\prime}_{2} \,\,\,$: Viscoelasticity of pure outer liquid $({\text{kg}}\,{\text{m}}^{ - 1} )$
$\mu_{1}$: Viscosity of pure inner liquid $({\text{kg}}\,{\text{m}}^{ - 1} \,{\text{s}}^{ - 1} )$
$\mu_{2}$: Viscosity of pure outer liquid $({\text{kg}}\,{\text{m}}^{ - 1} \,{\text{s}}^{ - 1} )$
$\varepsilon_{m}^{(1)}$: Magnetic permeability of pure inner liquid
$\varepsilon_{m}^{(2)}$: Magnetic permeability of pure outer liquid
$\kappa_{1}$: Thermal conductivity of pure inner liquid $({\text{W}}\,{\text{m}}^{ - 1} \,{\text{K}}^{ - 1} )$
$\kappa_{2}$: Thermal conductivity of pure outer fluid $({\text{W}}\,{\text{m}}^{ - 1} \,{\text{K}}^{ - 1} )$
$\eta$: Radius of dust particle (${\text{m}}$)
$$\zeta$$: Displacement disturbance $$({\text{m}})$$
$\varphi$: Velocity potential function $({\text{m}}^{2} \,{\text{s}}^{ - 1} )$
$\psi$: Magnetic potential function
$\sigma$: Relaxation time factor of dust particles
$\gamma$: Amplitude of the wave train solution
