# Supplementary material for: Nonlinear stability of two dusty magnetic liquids surrounded via a cylindrical surface: impact of mass and heat spread
Source: Sci Rep. 2023 May 1;13:7096. doi: 10.1038/s41598-023-33025-1 (PMC10151379; doi:10.1038/s41598-023-33025-1)
Supplement: Supplementary file 1 — Supplementary Information. [file 41598_2023_33025_MOESM1_ESM.pdf]

Appendix

The coefficients of Eq. (25-27) may be listed as follows:

$$g(l_1,l_2)=I_1(kl_1)K_1(kl_2)-I_1(kl_2)K_1(kl_1),$$
$$h(l_1,l_2)=I_0(kl_1)K_1(kl_2)+I_1(kl_2)K_0(kl_1),$$
$$\Lambda=(k\varepsilon_m^{(2)}h(r_1,R)g(r_2,R)-k\varepsilon_m^{(1)}h(r_2,R)g(r_1,R))+ik(\varepsilon_m^{(2)}-\varepsilon_m^{(1)})(h(r_1,R)h(r_2,R)+g(r_1,R)g(r_2,R))\zeta_z$$
$$+k(h(r_1,R)g(r_2,R)\varepsilon_m^{(1)}-h(r_2,R)g(r_1,R)\varepsilon_m^{(2)})\zeta_z^2.$$

The coefficients of Eq. (29) may be listed as follows:

$$A_1=((\rho_2+\mu_2')h(r_2,R)g(r_1,R)-(\rho_1+\mu_1')h(r_1,R)g(r_2,R))/(kg(r_1,R)g(r_2,R)),$$
$$A_2=(U_{02}(\rho_2+\mu_2')h(r_2,R)g(r_1,R)-U_{01}(\rho_1+\mu_1')h(r_1,R)g(r_2,R))/(kg(r_1,R)g(r_2,R))$$
$$A_3=\mu_2(2K_2N_2+\mu_2)h(r_2,R)/(k(K_2N_2+\mu_2)g(r_2,R))-\mu_1(2K_1N_1+\mu_1)h(r_1,R)/(k(K_1N_1+\mu_1)g(r_1,R))$$
$$+\alpha_1(\rho_1\mu_2'h(r_2,R)g(r_1,R)-\rho_2\mu_1'h(r_1,R)g(r_2,R)+g(r_1,r_2)h(R,R)\rho_1\rho_2)/(k\rho_1\rho_2g(r_1,R)g(r_2,R))$$
$$+i((\rho_2U_{02}h(r_2,R)/g(r_2,R))-(\rho_1U_{01}h(r_1,R)/g(r_1,R))),$$

$$A_4=\left(iH_0^2(\varepsilon_m^{(1)}-\varepsilon_m^{(2)})^2h(r_1,R)h(r_2,R)\right)/\left(h(r_1,R)g(r_2,R)\varepsilon_m^{(2)}-h(r_2,R)g(r_1,R)\varepsilon_m^{(1)}\right)$$
$$-U_{01}h(r_1,R)(\mu_1(2K_1N_1+\mu_1)+ik\rho_1U_{01}(K_1N_1+\mu_1))/\left(k(K_1N_1+\mu_1)g(r_1,R)\right)$$
$$+U_{02}h(r_2,R)(\mu_2(2K_2N_2+\mu_2)+ik\rho_2U_{02}(K_2N_2+\mu_2))/\left(k(K_2N_2+\mu_2)g(r_2,R)\right),$$
$$A_5=\frac{T}{R^2}-K_1N_1\alpha_1\mu_1h(r_1,R)/k\rho_1(K_1N_1+\mu_1)g(r_1,R)+K_2N_2\alpha_1\mu_2h(r_2,R)/k\rho_2(K_2N_2+\mu_2)g(r_2,R)-\alpha_1\mu_1h(r_1,R)/k\rho_1g(r_1,R$$
$$+\alpha_1\mu_2h(r_2,R)/k\rho_2g(r_2,R)-i(\alpha_1U_{01}h(r_1,R)/g(r_1,R)-\alpha_1U_{02}h(r_2,R)/g(r_2,R)).$$

The coefficients of Eq. (32) may be listed as follows:

$$a_0=(h(r_1,R)/k\,g(r_1,R))(\rho_1+\mu_1'/k_1)-(h(r_2,R)/k\,g(r_2,R))(\rho_2+\mu_2'/k_1),$$
$$a_1=(h(r_2,R)/\,g(r_2,R))(2\rho_2+\mu_2'/k_1)U_{02}-(h(r_1,R)/\,g(r_1,R))(2\rho_1+\mu_1'/k_1)U_{01},$$
$$b_1=(h(r_1,R)/k\,\rho_1g(r_1,R)(K_1N_1+\mu_1/k_1))((\mu_1\rho_1/k_1)(2K_1N_1+\mu_1/k_1)+\alpha_1(K_1N_1+\mu_1/k_1)(\rho_1+\mu_1'/k_1))$$
$$-(h(r_2,R)/k\,\rho_2g(r_2,R)(K_2N_2+\mu_2/k_1))((\mu_2\rho_2/k_1)(2K_2N_2+\mu_2/k_1)+\alpha_1(K_2N_2+\mu_2/k_1)(\rho_2+\mu_2'/k_1)),$$

$$a_2=(1/R^2-k^2)T-(h(r_1,R)/k\,\rho_1g(r_1,R))((\alpha_1\mu_1(2K_1N_1+\mu_1/k_1)/k_1(K_1N_1+\mu_1/k_1))-k^2\rho_1^2U_{01}^2)$$
$$+(h(r_2,R)/k\,\rho_2g(r_2,R))((\alpha_1\mu_2(2K_2N_2+\mu_2/k_1)/k_1(K_2N_2+\mu_2/k_1))-k^2\rho_2^2U_{02}^2)$$
$$+((kH_0^2(\varepsilon_m^{(1)}-\varepsilon_m^{(2)})^2h(r_1,R)h(r_2,R))/(h(r_2,R)g(r_1,R)\varepsilon_m^{(1)}-h(r_1,R)g(r_2,R)\varepsilon_m^{(2)})),$$
$$b_2=(\mu_2U_{02}h(r_2,R)(2K_2N_2+\mu_2/k_1)/k_1\,g(r_2,R)(K_2N_2+\mu_2/k_1))-(\mu_1U_{01}h(r_1,R)(2K_1N_1+\mu_1/k_1)/k_1\,g(r_1,R)(K_1N_1+\mu_1/k_1))$$
$$-\alpha_1U_{01}(h(r_1,R)/g(r_1,R))+\alpha_1U_{02}(h(r_2,R)/g(r_2,R)).$$
